# How old are you, really? Communicating chronic risk through ‘effective age’ of your body and organs

**DOI:** 10.1186/s12911-016-0342-z

**Published:** 2016-08-05

**Authors:** David Spiegelhalter

**Affiliations:** Winton Professor for the Public Understanding of Risk, Statistical Laboratory, Centre for Mathematical Sciences, Wilberforce Road, Cambridge, CB3 0WB UK

**Keywords:** Risk communication, Real age, Heart age, Lung age, Hazard ratio

## Abstract

In communicating chronic risks, there is increasing use of a metaphor that can be termed ‘effective-age’: the age of a ‘healthy’ person who has the same risk profile as the individual in question. Popular measures include ‘real-age’, ‘heart-age’, ‘lung-age’ and so on.

Here we formally define this concept, and illustrate its use in a variety of areas. We explore conditions under which the years lost or gained that are associated with exposure to risk factors depends neither on current chronological age, nor the period over which the risk is defined. These conditions generally hold for all-cause adult mortality, which enables a simple and vivid translation from hazard-ratios to years lost or gained off chronological age. Finally we consider the attractiveness and impact of this concept.

Under reasonable assumptions, the risks associated with specific behaviours can be expressed in terms of years gained or lost off your effective age. The idea of effective age appears a useful and attractive metaphor to vividly communicate risks to individuals.

**Electronic supplementary material:**

The online version of this article (doi:10.1186/s12911-016-0342-z) contains supplementary material, which is available to authorized users.

## Background

Communicating statistical risk information plays a vital part in the process of shared-care and informed health choices, whether using patient decision-aids or in more informal dialogue with health professionals. There has been recent guidance on communication tools for the risks of treatment outcomes [1], but these measures are concerned with the chances of events occurring within a fixed time period, say death following surgery.

Communicating chronic risks, associated with an increased chance event of an adverse event throughout the whole life-course, is more complex. The standard measure used by epidemiologists is the ‘hazard ratio’, which is the increased instantaneous risk associated with exposure to the risk factor, but this is a ‘relative risk’ measure that is known to produce an exaggerated impression [1].

An increasingly popular alternative is to assess what we shall call the ‘effective age’ of either a whole person or a particular organ. Many different terms are used for this metaphor: for example, websites will tell you your ‘real age’ [2] your ‘health age’ [3], ‘vitality age’ [4], or ‘biological age’ [5] (although the basis for these calculations is unclear), while your ‘Ubble Age’ [6] is that of people of the same gender whose mean risk of dying in the next 5 years matches your risk. You can also, if you wish, obtain your ‘heart age’ [7], ‘lung age’ [8], or ‘brain age’ [9].

In each case an individual enters various characteristics of their health and habits, and their effective age is calculated. The idea is to allow people to compare their effective age with their chronological age, and hence provide a vivid idea of their state of health. One aim is to motivate them to reduce an increased effective age by either changing their behaviour or taking medical interventions.

Here we define precisely what is meant by effective age, give examples of its use as a communication tool, and relate it to previous work on ‘risk advancement periods’. The difference between an individual’s effective and chronological age is an attractive measure of either premature ageing or continued youthfulness, and we derive conditions under which this difference is independent of both chronological age and the horizon over which the risk is measured. Finally, we summarise experience of the impact of this metaphor, and consider its future use.

## Main text

### The meaning of ‘effective age’

Given a specified measure of ‘risk’, your effective age can be defined as the age of a typical ‘healthy’ person who matches your risk profile. So if your chronological age is 50, but your effective age is 60, this means that you are in the same risk category as a 60-year old who has ‘healthy’ risk factors, or at least the ones that are potentially modifiable.

**Fig. 1:**
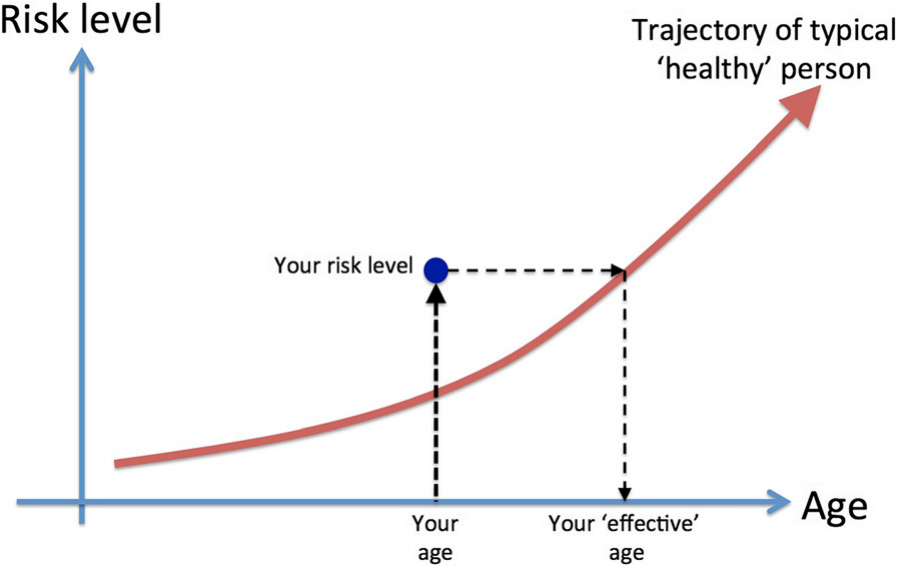
Schematic representation of the meaning of your ‘effective’ age with respect to a particular risk measure

The generic idea is shown in Fig. 1: a reference trajectory of a typical ‘healthy’ person is calculated, and then a subject’s actual risk level is mapped across to the find the age of a ‘healthy’ person with the matching level.

The same idea can be used for specific organs of the body. In particular, many cardio-vascular risk calculators will provide your ‘heart age’ or a ‘vascular age’ [10], and examples use a variety of risk measures:

- The UK National Health Service Heart Age [7], based on the JBS3 calculator [11]: this is the age of a someone of the same gender who has healthy risk factors and a matching annual risk of heart attack or stroke.
- The UK Heart Age calculator [12] and QRISK2 [13] calculate the age of a person with healthy risk factors and the same 10-year cardiovascular risk (assuming no deaths from other causes during this period).
- The New Zealand ‘Know your Numbers’ [14] program provides the age of someone with matching 5-year cardiovascular risk (again assuming no deaths from other causes during this period).

The concept of lung-age was developed over 30 years ago [15]: using updated equations, calculators [8] derive the age for which your lung function results would be expected in a typical person of your age and height.

There are two crucial decisions in defining an effective age. The first is the specification of a ‘healthy’ person: for example, the Heart Age calculator on NHS Choices [7] uses a person of the same gender and ethnicity, and who is a non-smoker with no clinical conditions, a BMI of 26, systolic blood pressure 120, and LDL/HDL cholesterol ratio of 3.5. This choice of a ‘standard’ individual can have a major impact on the properties of the procedure. The ‘Ubble age’ calculator [6], based on nearly 500,000 UK Biobank participants [16], uses the population 5-year all-cause mortality as the reference level, but most of this risk comes from the few individuals who are already sick, and so greatly overestimates the risk of the vast majority of the population: it is one of those anomalous situations where nearly everyone is less than average. The consequence is that almost everyone’s Ubble age is considerably less than their chronological age – often around 10 years. In this case a better comparator may have been the 5-year all-cause mortality of a healthy population, or the median rather than mean risk.

The second decision is the choice of risk measure, and in particular whether it represents an instantaneous risk or ‘hazard’, or is defined as the chance of an adverse event of a fixed horizon, say 10 years. There is a strong relation to the work of Brenner and colleagues [17], who consider the difference between a person’s effective age (although they do not use this term) and their chronological age as advancing the time that they may suffer an adverse event, which they call an ‘advancement period’. If the risk is an ‘instantaneous’ measure, they term this a ‘rate advancement period’, and if it is a risk over a fixed horizon, say the chance of developing cancer in the next 10 years, they term this a ‘risk advancement period’.

For example, Liese and colleagues [18] used Rate Advancement Periods (RAPs) to communicate increased heart-attack risk from hypertension (RAP = 8 years) and smoking (RAP =11 years): the latter says that, compared with never/former smokers, smokers are expected to advance their risk of myocardial infarction approximately 11 years.

### When can we ignore your current age and the risk horizon?

The difference between effective and chronological age could be called the ‘years lost/gained’ - for example if you are 50 years old, but your effective age is 60, you have ‘lost’ 10 years. It would be attractive if this difference did not depend on chronological age, so individuals could be told how specific behaviours added or subtracted so many years from their effective age: for example, that smoking 20 a day added 8 years to your effective age, however old you are at the moment.

Suppose we are concerned with a risk measure *R*, a chronological age *t*, and an exposure *x*, coded 1 if exposed, 0 otherwise. Then Brenner et al. [17] show that if there is an increasing function *g* such that

$$ g(R)=a+bx+ct, $$

then an individual with the risk factor (*x* = 1) and chronological age *t*_0_ will have the same risk as someone without the risk factor (*x* = 0) and age *t*_1_ = *t*_0_ + *b*/*c*, since

1 $$ g(R)=a+b+c{t}_0=a+c\left({t}_0+b/c\right) $$

Thus under these conditions the ‘risk advancement period’ *t*_1_ − *t*_0_ = *b*/*c* does not depend on the chronological age *t*_0_.

We’ve also seen that, for example, different versions of ‘heart-age’ can depend on your risk of a heart attack this year, over the next 5 years, or the next 10 years. It would be desirable if this risk ‘horizon’ were irrelevant.

It turns out that there are reasonable assumptions under which both your years lost/gained do not depend on your current age, and your effective age does not depend on the risk horizon. In the Additional file 1 we prove that both these attractive properties hold given the following two conditions:

- Condition 1. There are ‘proportional hazards’: i.e. risk factors act on the baseline trajectory by increasing the annual risk by a percentage that does not depend on current age.
- Condition 2. The instantaneous risk (hazard) of a healthy person increases exponentially with age, i.e. each year increases the risk by a fixed percentage.

These conditions are equivalent to assuming, in Eq. (1), that *h* is the instantaneous risk of an event, and *g* is the logarithmic function, so that *log*(*h*) = *a* + *bx* + *ct*, or

2 $$ h={e}^{a+bx+ct} $$

Thus *e*^*b*^ and *e*^*c*^ are the hazard ratios associated with a unit increase in the risk factor, and a year of ageing, respectively.

Fortunately these two conditions are reasonably plausible in a wide range of situations. The first condition is the standard assumption in the Cox regression model used widely in epidemiological studies, while we now show that the second condition of an exponentially-increasing hazard can be assumed to hold over a wide age-range, at least for all-cause mortality.

**Fig. 2:**
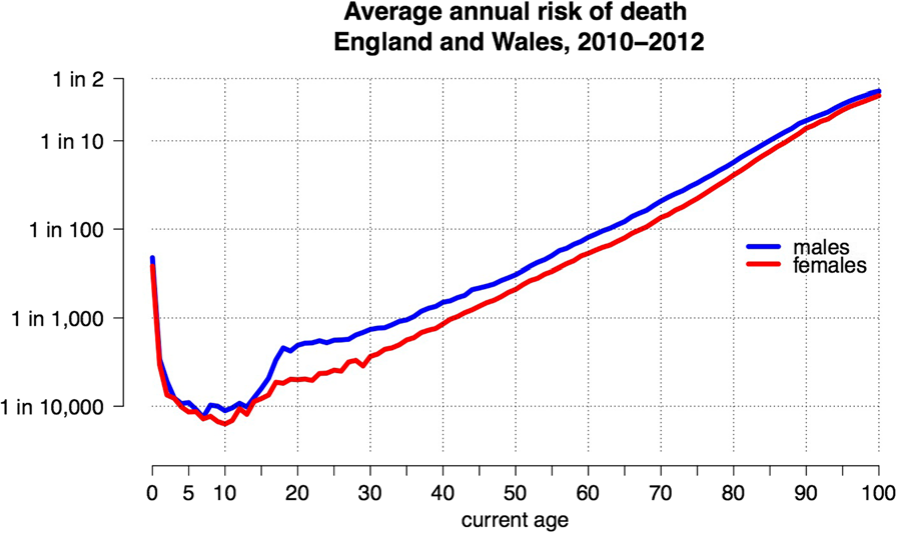
Annual risk of death from all causes for England and Wales, 2010–2012, known as the annual ‘hazard’ or ‘force of mortality’

Figure 2 shows the annual risk (or hazard) of death for an average person during each year of age in England and Wales between 2010 and 2012 [19] – this is traditionally known as the ‘force of mortality’.

We note the steep decline once one has survived early childhood, to a minimum of less than 1 in 10,000 at around 10 years old (in spite of the anxieties expressed about vulnerable children, nobody in the history of humanity has been as safe as a modern Western 10-year-old). Then, apart from a ‘risk-taking’ bulge between 15 and 25, particularly in young men, there is a reasonably straight line until around 95. This linear growth on a logarithmic scale corresponds to an exponential growth in annual hazard, meaning the risk of dying each year increases by a fixed proportion for each year we age, exactly the condition we seek.

**Fig. 3:**
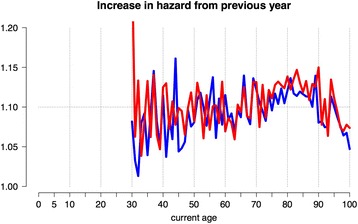
Between ages 30 to 100, the year-on-year increase in annual all-cause mortality risk for England and Wales, 2010–2012

We can check this observation by plotting the ratio of hazards for adjacent years (Fig. 3), which shows that the relative increase is fairly constant between ages 30 and 95 – this observation that dates back to Benjamin Gompertz in 1825 [20]. The increase in annual hazard associated with ageing 1 year is roughly around 1.1, which is *e*^*c*^ in our previous notation - this means that the average chance of dying before a next birthday increases by around 10 % for each year of ageing, whether a man or a woman and regardless of age, if over 30. Equivalently, the average risk of dying before a next birthday doubles roughly every 7 years. Demographers have concluded that this ratio, 1.1, seems to be remarkably constant across populations and over time [21].

### The relationship between specific behaviours and effective age

We now show that, given the two conditions of proportional and exponentially increasing hazards, we obtain an elegantly simple way of converting the estimated effects associated with different exposures to years of life lost or gained.

Standard output from epidemiological studies includes the estimated all-cause mortality hazard ratio, which is the increased annual risk of death for those with the risk factor compared to those without. The estimates are derived from Cox regression and apply over the range of ages being studied. The second column of Table 1 shows some examples from recent epidemiological studies.

**Table 1:** Hazard ratios (r) associated with specific behaviours derived from recent epidemiological studies, translated into ‘changes in effective age’ (t) through the formula *t* ≈ 10 ln(*r*)

*Exposure/behaviour*	*Estimated hazard ratio r*	*Change in effective age if behaviour present (years)*	*Reference*
Smoking 20 cigarettes a day	2.20	+8	[30]
Eating 50 g processed meat a day	1.18	+2	[31]
Watching 2 h of TV a day	1.08	+1	[32]
An extra 5 units of BMI (Kg/m2) above 25.	1.29	+3	[33]
Minimal exercise (compared to inactivity)	0.82	−2	[34]
Further exercise (compared to minimal)	0.92	−1	[34]
Eating fruit and vegetables (per 2 portions a day)	0.90	−1	[35]
Taking statins (higher-risk patients)	0.91	−1	[36]

The crucial insight is that these hazard ratios can be easily translated into changes in effective age. Suppose *r* is the hazard ratio associated with a risk factor, where *r* = *e*^*b*^ and so *b* is the log(hazard ratio) obtained in a Cox regression analysis. The second condition says that each increased year of age is associated with a hazard ratio *e*^*c*^, and so the increased risk associated with the risk factor is equivalent to a change of *t* years in your effective age if

$$ {e}^{ct}=r={e}^b $$

or equivalently

$$ t=\frac{b}{c}=\frac{ \ln (r)}{c}, $$

the result derived in Eq. (1). Since *c* ≈ ln(1.1) ≈ 0.1, we have that *t* ≈ 10 ln(*r*). Applying this formula to the risk factors in Table 1, and rounding to the nearest integer, provides the values in the column headed ‘*Change in effective age*’. Translating each risk factor into a change in effective age provides a simple, but fairly rigorous, form of communication: for example, 2 h TV a night is associated with the same increased mortality risk as if you were 1 year older, for any age for which this hazard ratio holds.

There are three important caveats. Confronted with Table 1, the immediate temptation is to start adding the effects of the relevant exposures to get an overall number of years lost or gained. This assumes the values are all valid simultaneously, which would be true, for example, if all the hazard ratios had been calculated from a single Cox regression analysis with no interactions, However, combinations of estimates from different studies may not be too misleading provided the exposures are clearly distinct. This means there could be a reasonably sound basis for the sort of ‘real-age’ calculations shown on websites, although in practice the numbers used may often be more guesswork than based on the latest epidemiology.

Second, there must also be caution over any causal interpretation and assumption of reversibility – we should not casually say that changing behaviour will ‘take years’ off your age unless based on randomised evidence.

Finally, while we have argued that these two conditions hold for all-cause mortality, organ-specific effective age calculators are derived from other metrics such as the risk of cardiovascular events in current heart age calculators. These calculators assume a proportional hazard model and so the first condition holds, and the annual risk of cardiovascular events, while not specifically assumed to be exponential, will be strongly correlated with the risk of all-cause mortality. Thus the two conditions should roughly hold, and so the shift in effective age should be approximately independent of chronological age.

As a check, raised risk factors entered into QRISK2 that raised a chronological age of 60 to a ‘QRISK Healthy Heart Age’ of 64 were found to produce a similar difference of either 3 or 4 years when the chronological age was varied between 30 and 80.

### The impact of communicating effective age

Concepts such as effective age are clearly attractive and even gripping metaphors: a journalist recently listed the ‘age’ of each of their organs [22], and it is claimed that 27,000,000 people have been provided with their Real Age.

There is also some evidence that these ideas might help change behaviour and improve risk factors. A recent review [10] claims that vascular age is easily understood by patients and has a greater impact on care than presenting an estimated cardio-vascular disease risk score, citing a study [23] in which Heart Age *“was more emotionally impactful in those participants at higher actual CVD risk levels”*. A trial [24] randomised over 3000 subjects to either conventional medical advice, a risk score or Heart Age, and found that levels of metabolic parameters had improved significantly after 12 months of follow-up in both risk communication groups, but more in the group randomised to receive their Heart Age. Heart age had increased in the control group and decreased in the intervention groups*.*

For ‘lung age’, the Step2quit study [25] found double the quit-rates in smokers who were told their lung age (6 % vs 14 %), and this led to recommendations [26] to routinely use this concept. However a study [27] of 144 smokers cast doubt on the ability of lung-age to increase motivation, while it’s been suggested that the concept may even *reduce* motivation to quit for those with a ‘normal’ lung age [28].

## Conclusions

Appropriate methods for communicating risk information is a subject of intense current interest [1], but attention tends to focus on risks expressed as simple probabilities of adverse events. Chronic risks that influence long-term outcomes are more complex. This points to the need for randomised trials of alternative methods of providing chronic risk information, for example in comparison with other metaphors such as hazard ratios, changes in life-expectancy, and time lost per exposure, for example losing 15 min off your life expectancy for each cigarette [29].

The idea of behaviours being associated with adding or subtracting years from your effective age is clearly attractive to many people, and we have shown that these quantities can be made rigorous under plausible assumptions. Concepts based on the idea of effective age, such as Heart Age, are likely to become an increasingly familiar part of the discourse around risk and behaviour.

## Abbreviations

JBS3, Joint British Societies’ Recommendations on the Prevention of Cardiovascular Disease (3^rd^ edition); RAP, rate advancement period

## Additional file

Additional file 1: Effective-age-appendix. Contains mathematical proofs of result in paper. (PDF 106 kb)
