# Supplementary material for: How old are you, really? Communicating chronic risk through ‘effective age’ of your body and organs
Source: BMC Med Inform Decis Mak. 2016 Aug 5;16:104. doi: 10.1186/s12911-016-0342-z (PMC4974726; doi:10.1186/s12911-016-0342-z)
Supplement: Additional file 1: — Effective-age-appendix. Contains mathematical proofs of result in paper. (PDF 106 kb) [file 12911_2016_342_MOESM1_ESM.pdf]

Appendix to *How old are you, really? Communicating chronic risk through effective age of your body and organs*, by David Spiegelhalter

Let T be the age at which an adverse event E happens to an individual. Let $S(t;x)$ the survivor function at age t for someone with risk factors x, i.e. $S(t;x) = P(T > t;x)$, the probability that the adverse event in question has not occurred by age t.

Suppose the 'risk level' r of a person aged t_1 with risk factors x_1 is defined as the probability of the event first occurring to them within the next k years. so that

$$r = P(t_1 < T < t_1 + k | T > t_1; x) = \frac{P(t_1 < T < t_1 + k; x)}{P(T > t_1; x)} = \frac{S(t_1; x) - S(t_1 + k; x)}{S(t_1; x)}.$$

Suppose a 'healthy' person has risk factors x_0. Then the effective age t_0 of the individual in question is the age at which a healthy person has risk r of experiencing the event in the next k years, and so, as before,

$$r = P(t_0 < T < t_0 + k | T > t_0; x_0) = \frac{P(t_0 < T < t_0 + k; x_0)}{P(T > t_0; x_0)} = \frac{S(t_0; x_0) - S(t_0 + k; x_0)}{S(t_0; x_0)},$$

and so

$$\frac{S(t_1; x_1) - S(t_1 + k; x_1)}{S(t_1; x_1)} = \frac{S(t_0; x_0) - S(t_0 + k; x_0)}{S(t_0; x_0)},$$

or equivalently

$$\frac{S(t_1 + k; x_1)}{S(t_1; x_1)} = \frac{S(t_0 + k; x_0)}{S(t_0; x_0)}. \tag{1}$$

Let $h(t;x)$ be the hazard function at age t for an individual with risk factors x, where h is the instantaneous risk of E occurring given it has not occurred up to age t. Thus $h(t;x) = p(t;x)/S(t;x)$, the cumulative hazard $H(t;x) = \int h = -\log S(t;x)$, and so $S(t;x) = e^{-H(t;x)}$.

Equation (1) can therefore be written in terms of the cumulative hazard as

$$H(t_1 + k; x_1) - H(t_1; x_1) = H(t_0 + k; x_0) - H(t_0; x_0). \tag{2}$$

We now introduce the first assumption of proportional hazards, which means that the hazard function can be expressed as $h(t;x) = h_0(t)e^{bx}$, where h_0 is a baseline hazard function, essentially that for $x = 0$. Thus $H(t;x) = H_0(t)e^{bx}$ for a cumulative baseline hazard H_0.

Equation (2) becomes

$$(H_0(t_1 + k) - H_0(t_1))\, e^{bx_1} = (H_0(t_0 + k) - H_0(t_0))\, e^{bx_0}. \tag{3}$$

or equivalently

$$\log\left(H_0(t_0+k)-H_0(t_0)\right)=\log\left(H_0(t_1+k)-H_0(t_1)\right)+b(x_1-x_0). \tag{4}$$

We now bring in the second assumption that the hazard is exponentially increasing, which we can express as $h_0(t) = de^{ct}$, the Gompertz equation. Then the proportional hazard assumption means that $h(t;x) = de^{bx}e^{ct}$, so the risk factors just have the effect of increasing the constant.

Given this assumption, we see that

$$H_0(t_1+k)-H_0(t_1)=\int_{t_1}^{t_1+k} h_0(t)dt = d\int_{t_1}^{t_1+k} e^{ct}dt = \frac{d}{c}e^{ct_1}\left[e^{ck}-1\right].$$

Substituting this in Equation (4) and cancelling terms gives

$$ct_1 = ct_0 + b(x_1-x_0), \tag{5}$$

or equivalently

$$t_1 = t_0 + \frac{b}{c}(x_1-x_0), \tag{6}$$

we immediately see that

- the lost years $t_1 - t_0$ does not depend on the current age,
- the effective age t_1 does not depend on k, and so the risk horizon is irrelevant.

We note that for binary exposures $x_1 - x_0 = 1$, and hence the 'years lost' is $\frac{b}{c}$: this is also the Rate Advancement Period. It can be estimated by fitting both the exposure and age as covariates in a Poisson regression, and then taking the ratio of their coefficients. Alternatively, in a Cox regression, exposure and age-at-entry can be fitted as covariates, and time-in-study used as the time variable.
